# Supplementary figures and images for: RNase E cleavage shapes the transcriptome of Rhodobacter sphaeroides and strongly impacts phototrophic growth
Source: Life Sci Alliance. 2018 Aug 1;1(4):e201800080. doi: 10.26508/lsa.201800080 (PMC6238624; doi:10.26508/lsa.201800080)

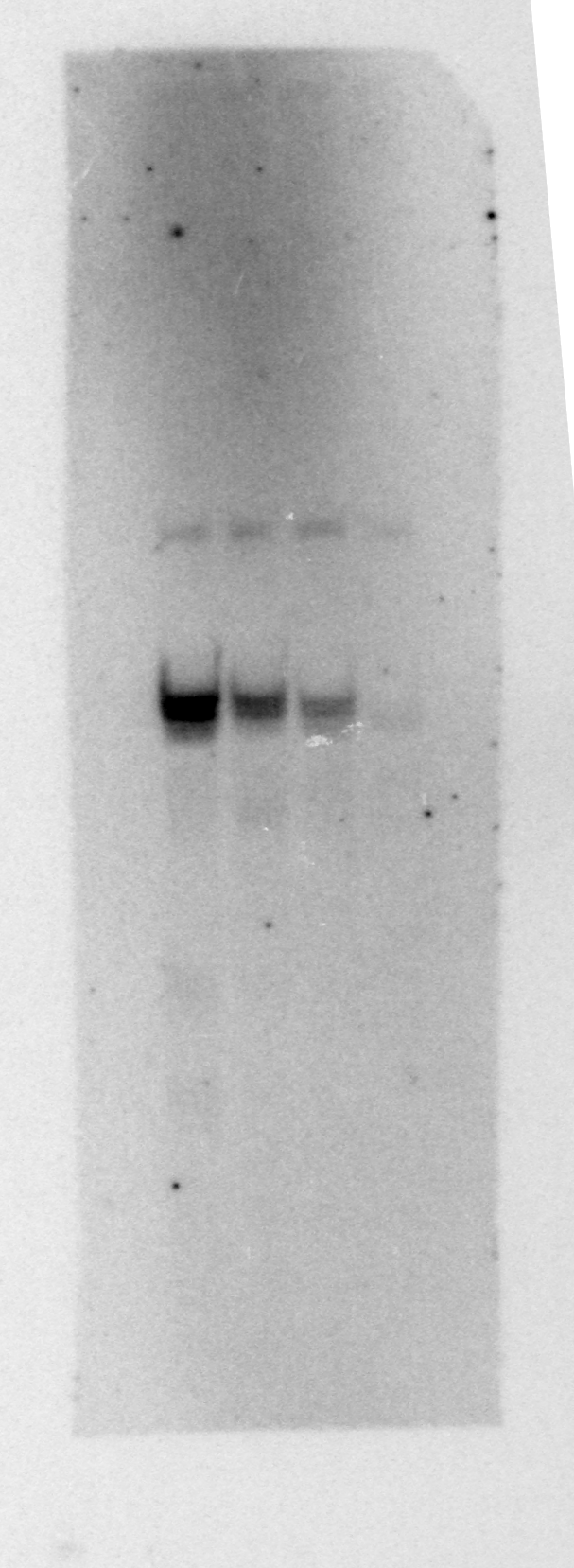

Supplement: Supplementary file 1 [file LSA-2018-00080__Preparation_of_compounds.zip › Northern_blot_sources/RSP_1771.tif]

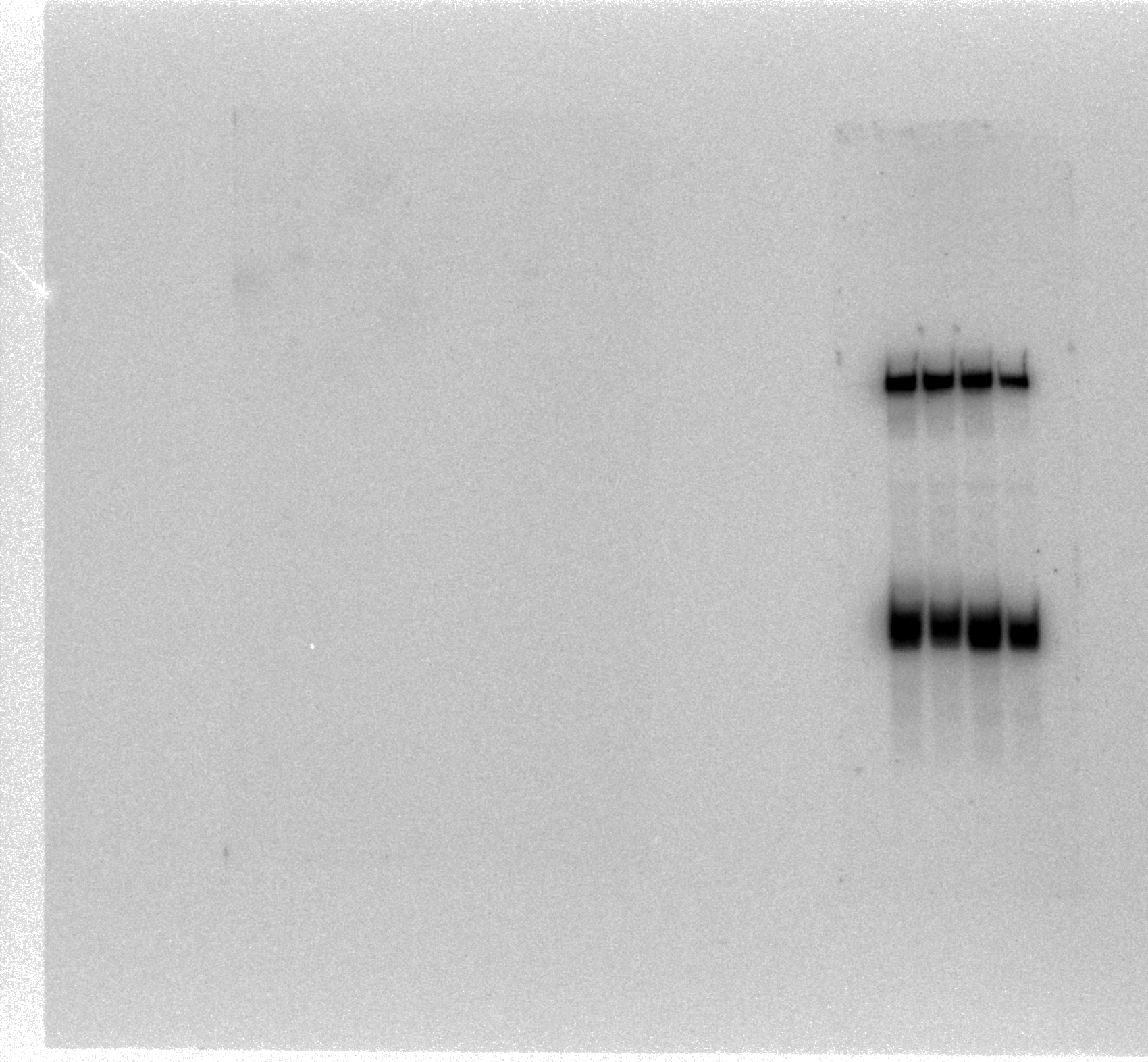

Supplement: Supplementary file 1 [file LSA-2018-00080__Preparation_of_compounds.zip › Northern_blot_sources/PcrZ.tif]

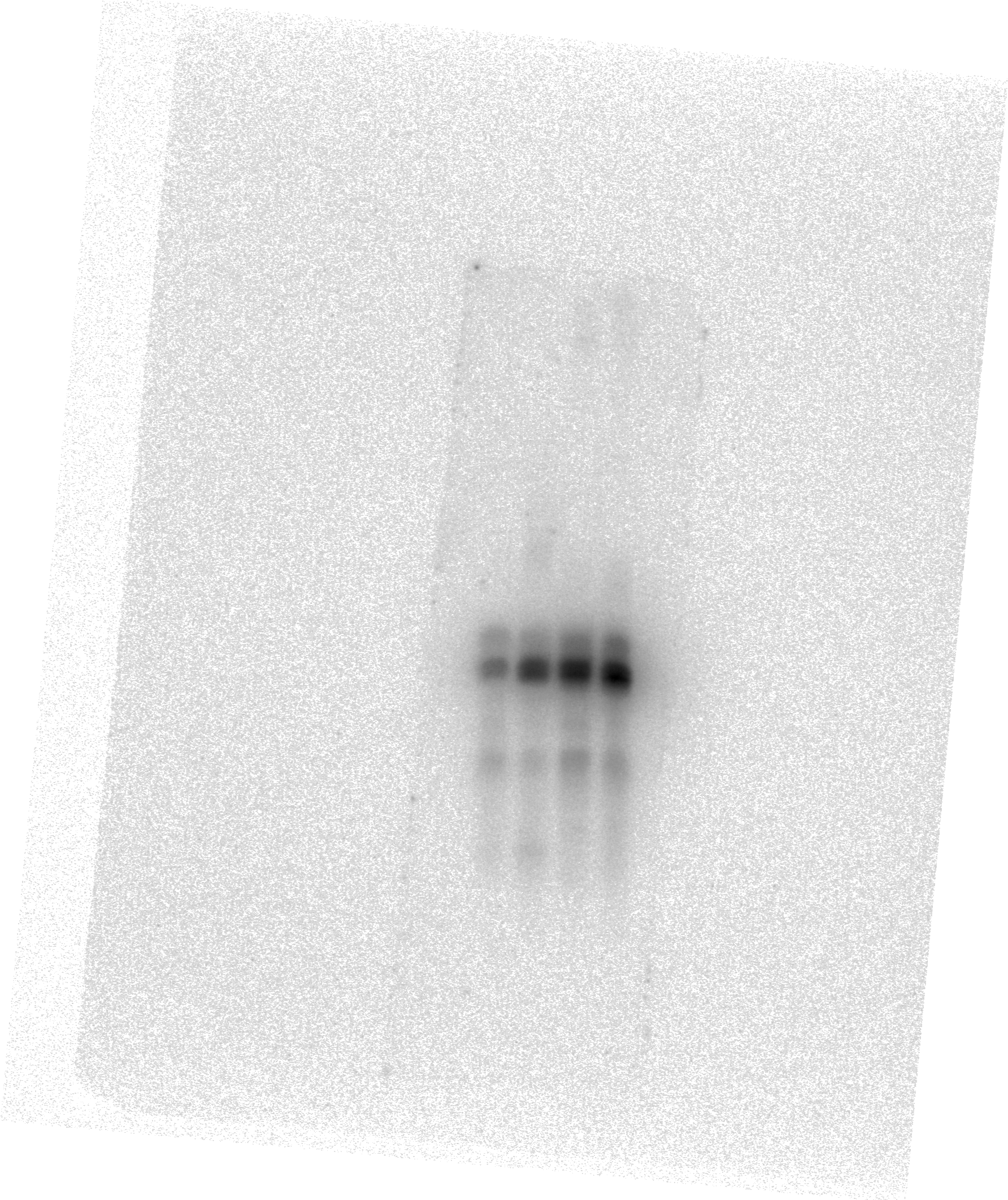

Supplement: Supplementary file 1 [file LSA-2018-00080__Preparation_of_compounds.zip › Northern_blot_sources/SorY.tif]

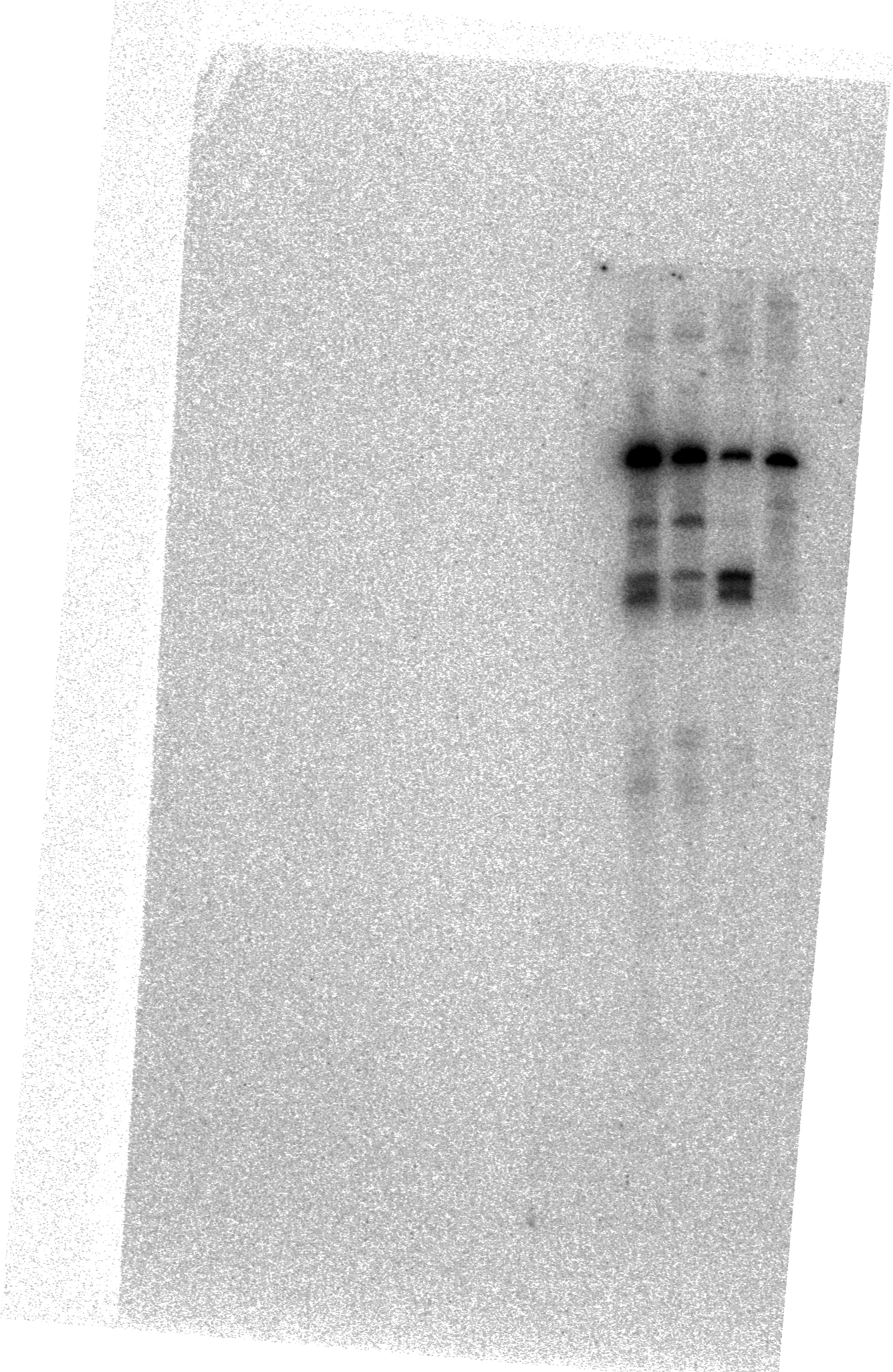

Supplement: Supplementary file 1 [file LSA-2018-00080__Preparation_of_compounds.zip › Northern_blot_sources/UpsM.tif]

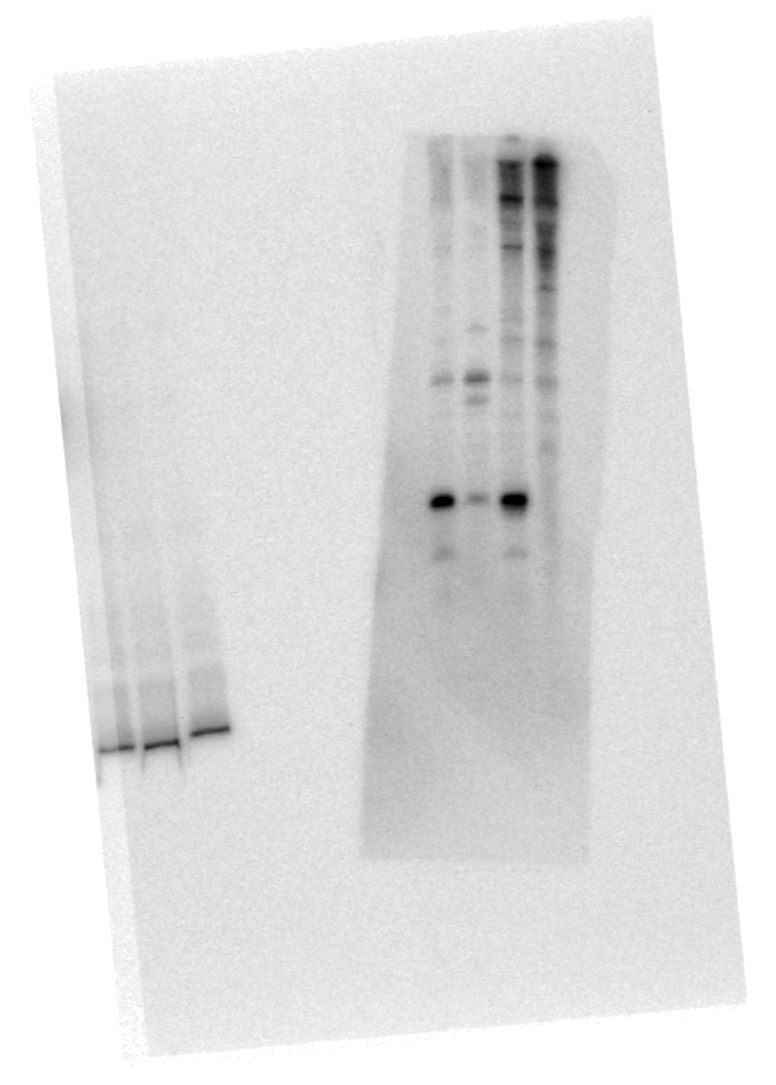

Supplement: Supplementary file 1 [file LSA-2018-00080__Preparation_of_compounds.zip › Northern_blot_sources/SorX.tif]

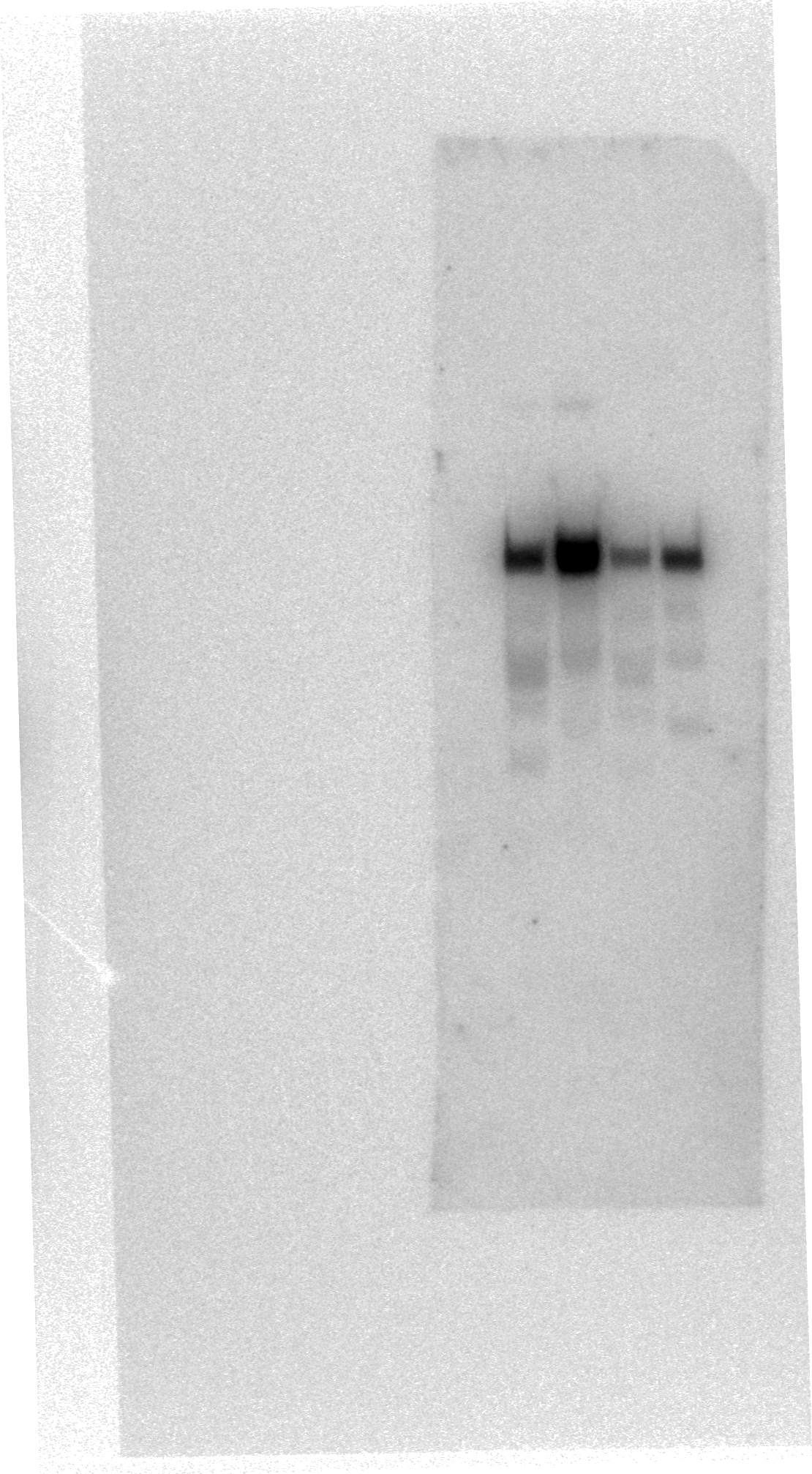

Supplement: Supplementary file 1 [file LSA-2018-00080__Preparation_of_compounds.zip › Northern_blot_sources/RSP_1624.tif]

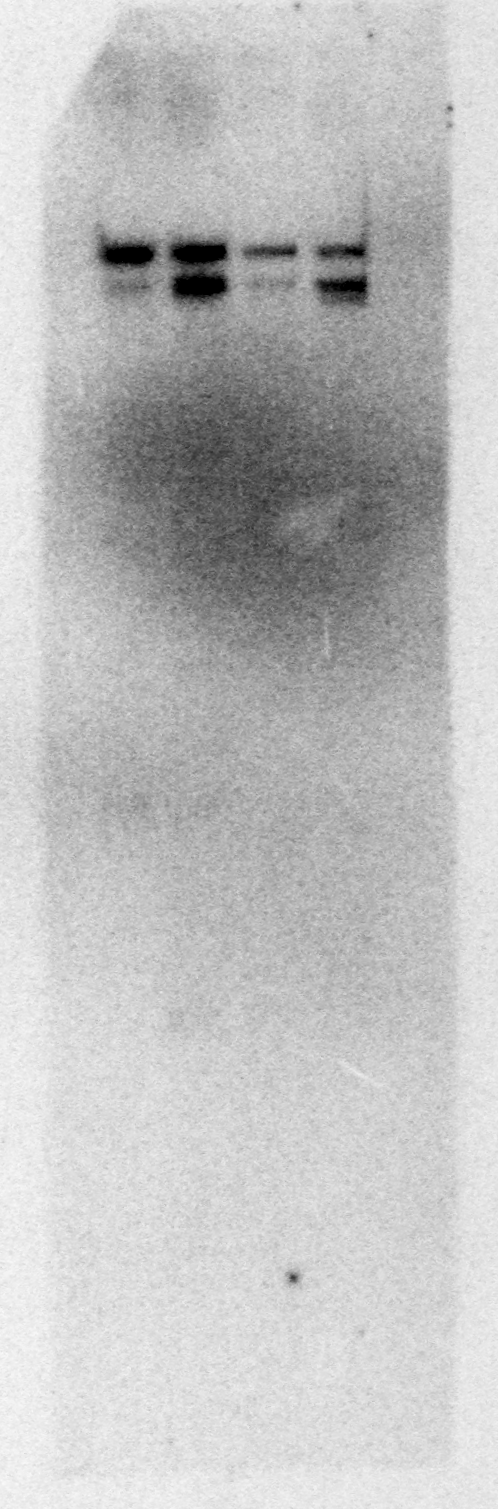

Supplement: Supplementary file 1 [file LSA-2018-00080__Preparation_of_compounds.zip › Northern_blot_sources/RSP_7517.tif]

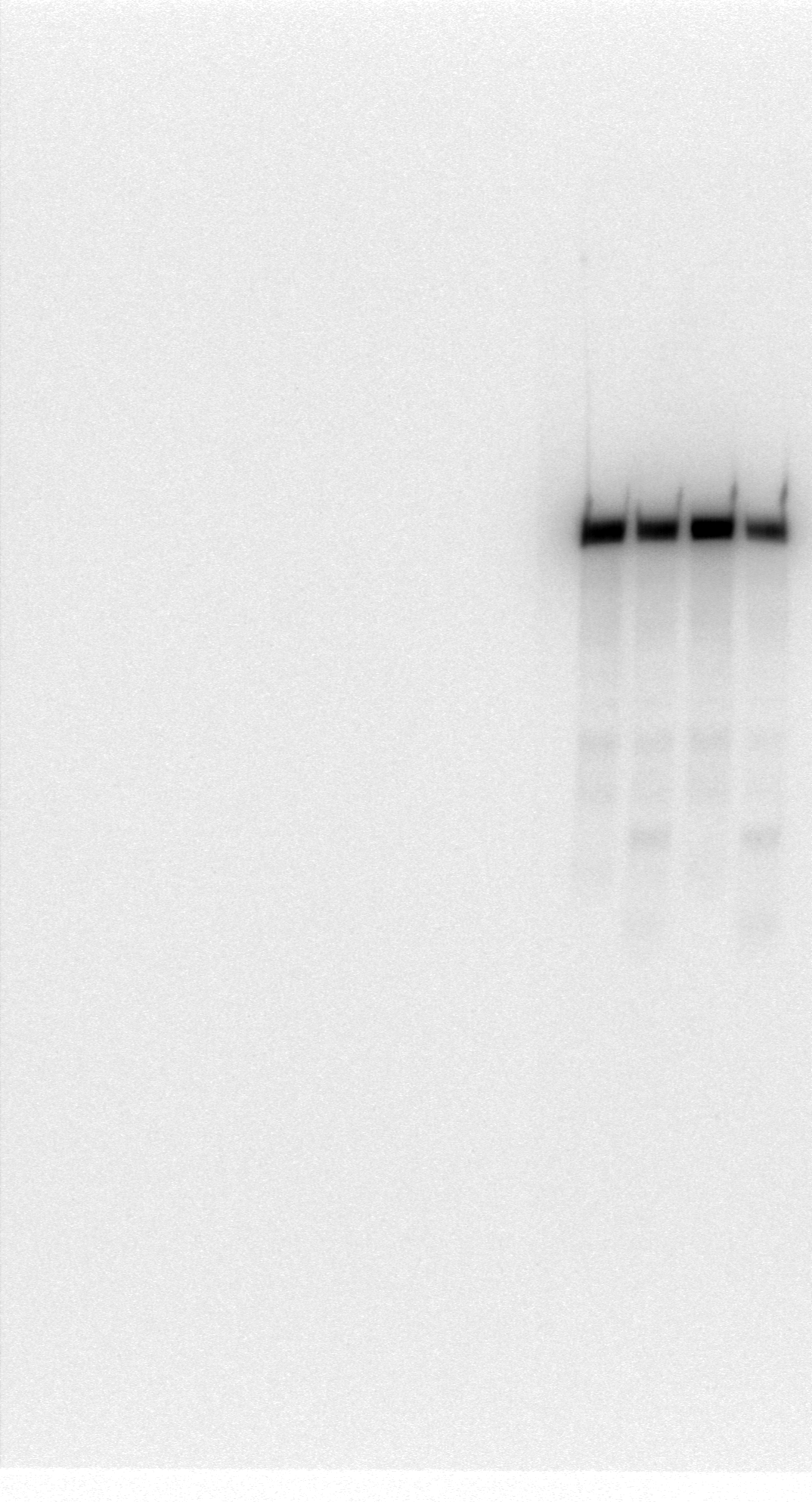

Supplement: Supplementary file 1 [file LSA-2018-00080__Preparation_of_compounds.zip › Northern_blot_sources/5S_for_RSP_7517.tif]

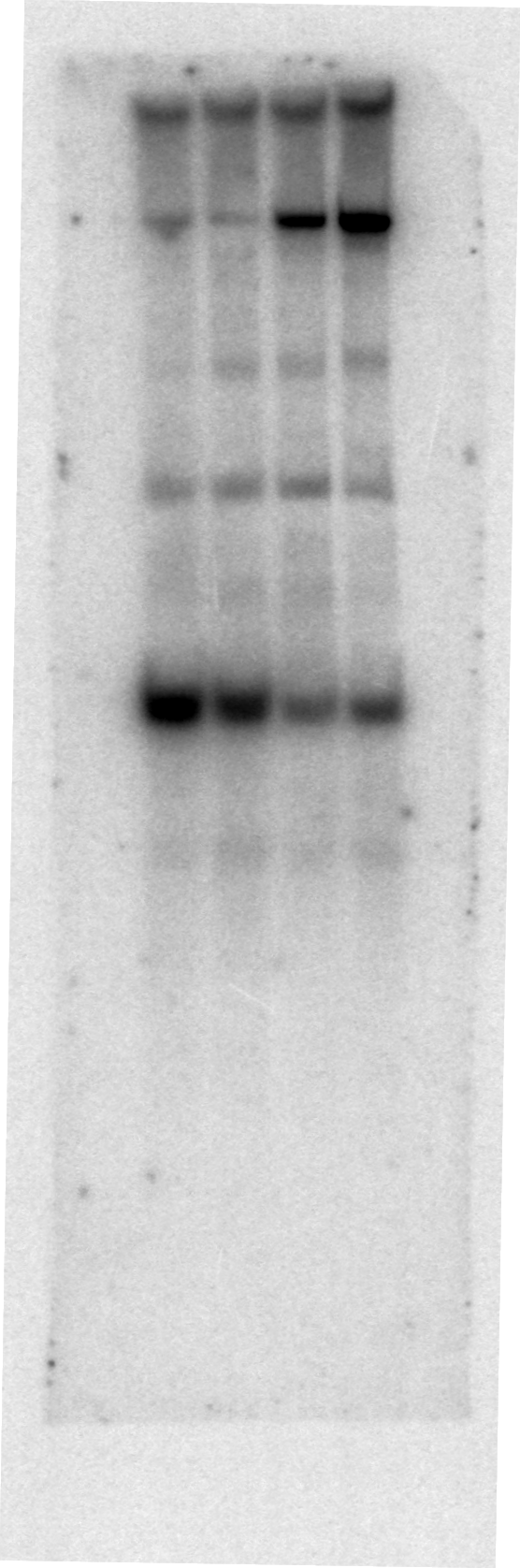

Supplement: Supplementary file 1 [file LSA-2018-00080__Preparation_of_compounds.zip › Northern_blot_sources/RSP_7527.tif]

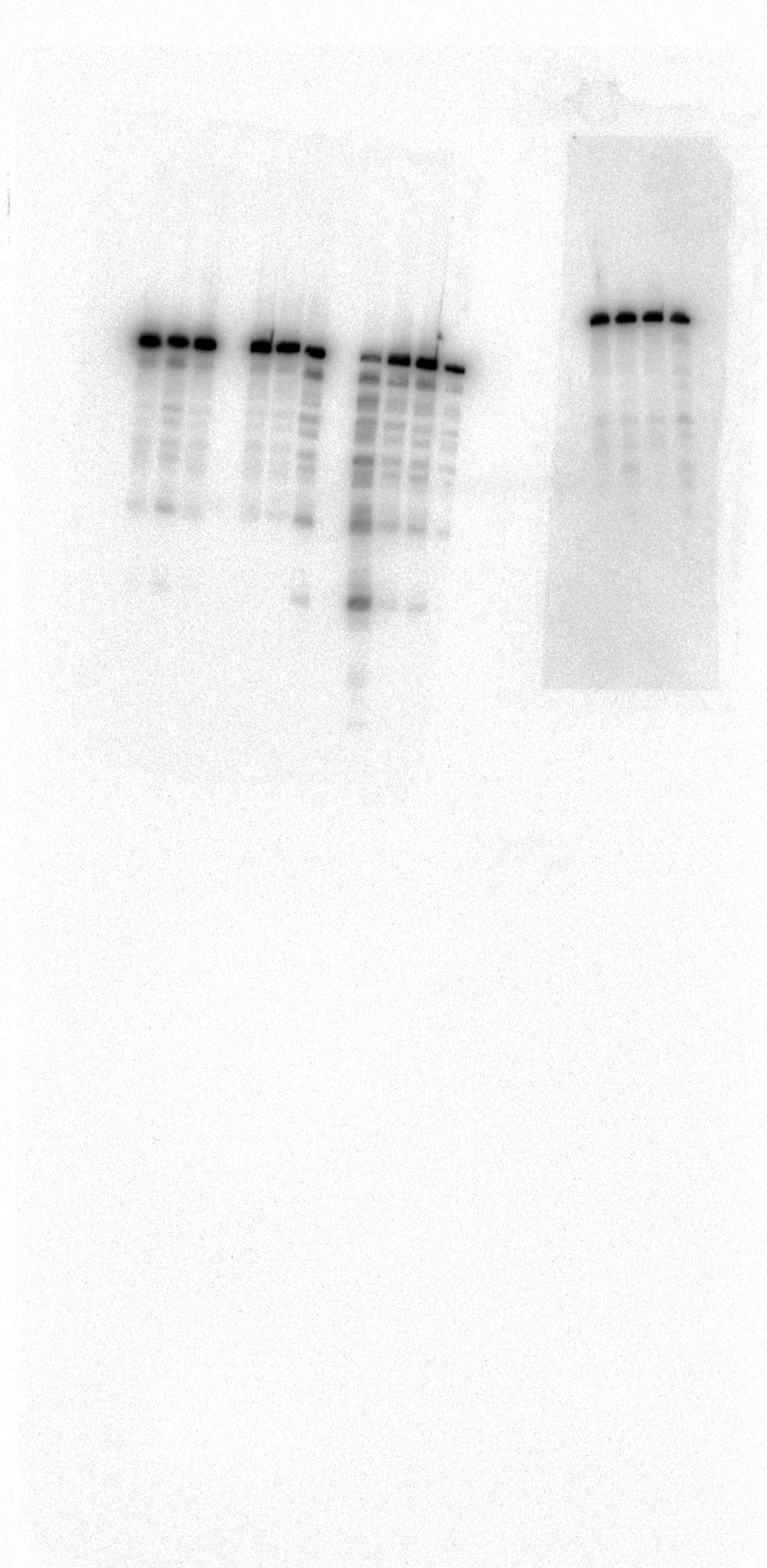

Supplement: Supplementary file 1 [file LSA-2018-00080__Preparation_of_compounds.zip › Northern_blot_sources/5S_for_SorX-SorY-UpsM.tif]

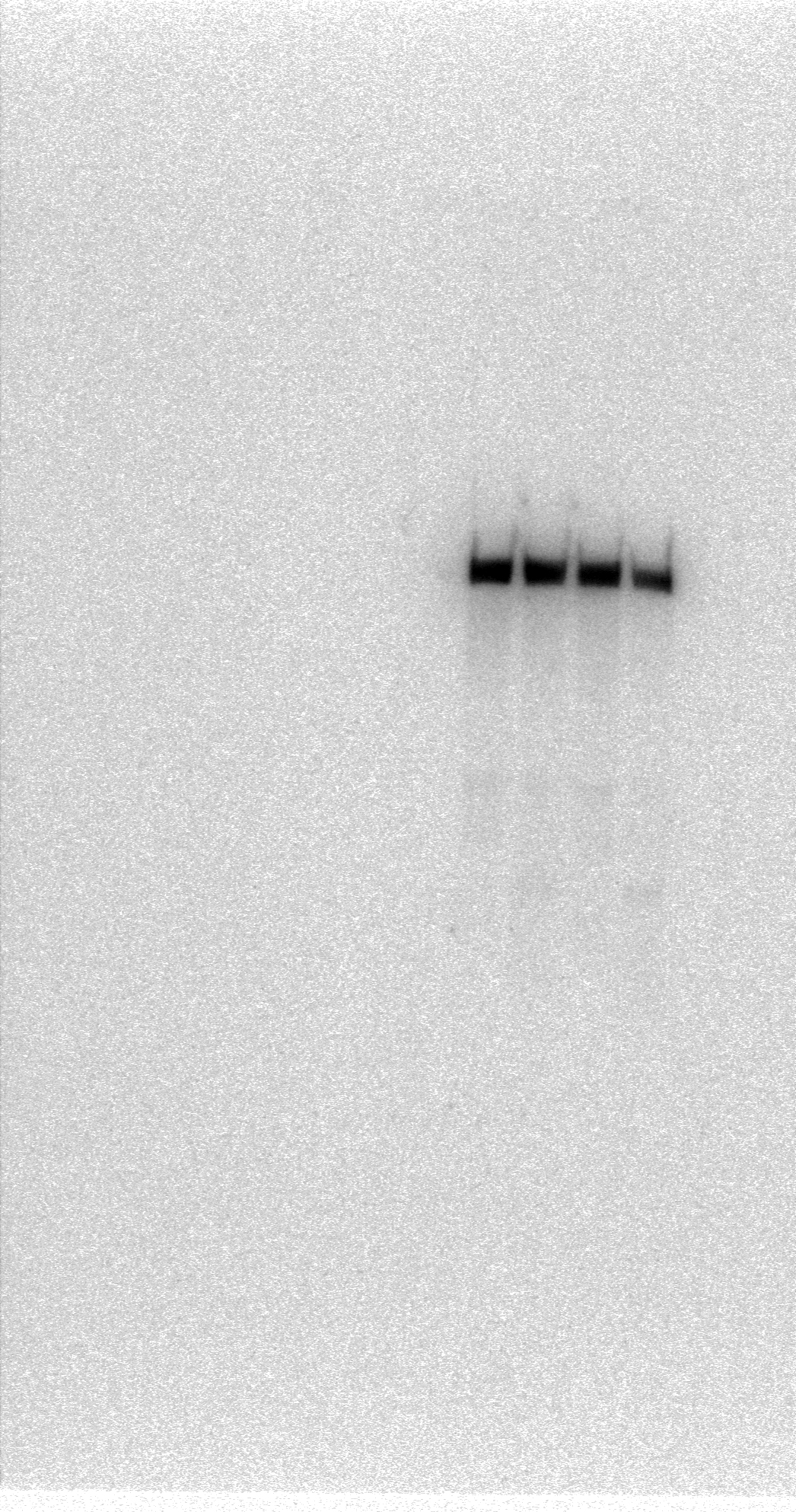

Supplement: Supplementary file 1 [file LSA-2018-00080__Preparation_of_compounds.zip › Northern_blot_sources/5S_for_RSP_0557-RSP_7527-RSP_1771-RSP_1624-PcrZ_.tif]
