# Supplementary material for: RNase E cleavage shapes the transcriptome of Rhodobacter sphaeroides and strongly impacts phototrophic growth
Source: Life Sci Alliance. 2018 Aug 1;1(4):e201800080. doi: 10.26508/lsa.201800080 (PMC6238624; doi:10.26508/lsa.201800080)
Supplement: Supplementary file 2 [file LSA-2018-00080_TableS1.pdf]

Table S1: Oligodeoxynucleotides used for hybridization.

Name	Sequence 5‘- 3‘
1771	CCAACTTACCGCAGCTCCGGTA
7517	CCGACGCTGAGGAACCTTCCTC
7527	TGTCTTGTCCATTCGTCCGGGTCA
0557	CACGACGGTGGCGAGGAGGTCG
UpsM	GACTCAGGTGGTCGCCAGATACC
SorY	ATGAAGCGGACGAGAGAACCCTC
1624	TTCTTGTCATAATTCGTTTCGTTATG
5S	CTTGAGACGCAGTACCATTG
SorX forward	TGTGATCGGCACGAACGAAG
SorX reverse	AAGGCTGGCACGAAGCAAGA
